# Association between lack of sexual interest and all-cause mortality in a Japanese general population: The Yamagata prospective observational study

**DOI:** 10.1371/journal.pone.0277967

**Published:** 2022-12-14

**Authors:** Kaori Sakurada, Tsuneo Konta, Narumi Murakami, Naoko Kosugi, Takafumi Saito, Masafumi Watanabe, Kenichi Ishizawa, Yoshiyuki Ueno, Takamasa Kayama

**Affiliations:** 1 Department of Fundamental Nursing, Yamagata University Graduate School of Nursing, Yamagata, Japan; 2 Department of Public Health and Hygiene, Yamagata University Graduate School of Medical Science, Yamagata, Japan; 3 Division of Nursing, Yamagata University Hospital, Yamagata, Japan; 4 Department of Clinical Nursing, Yamagata University Graduate School of Nursing, Yamagata, Japan; 5 Global Center of Excellence, Yamagata University School of Medicine, Yamagata, Japan; Weill Cornell Medical College in Qatar, QATAR

## Abstract

**Background:**

Sexual interest is essential for maintaining positive sexual relationships and sexual function, which have recently been recognized as important indicators of good health and quality of life. Here, we prospectively investigated associations between sexual interest and mortality in a community-based population.

**Methods:**

This study enrolled 20,969 subjects (8,558 males and 12,411 females) aged ≥ 40 years who participated in annual health check-ups in Yamagata Prefecture. Sexual interest was assessed by a self-report questionnaire. Associations between sexual interest and increased all-cause mortality, cardiovascular disease mortality, and cancer mortality were investigated by Cox proportional hazards modeling.

**Results:**

During follow-up (median: 7.1 years), 503 subjects died; 67 deaths were due to cardiovascular disease, and 162 were due to cancer. Kaplan-Meier analysis showed that all-cause mortality and cancer mortality were significantly elevated among men who lacked sexual interest (log-rank *P*<0.0001, *P*<0.05). Cox proportional hazards model analysis with adjustment for age, hypertension, diabetes, dyslipidemia, smoking, alcohol drinking status, BMI, education, marital status, frequency of laughter, and psychological distress showed that the risk of all-cause mortality was significantly higher among men who lacked sexual interest than men who had sexual interest (hazard ratio [HR] 1.69; 95% confidence interval [CI], 1.17–2.44).

**Conclusion:**

Lack of sexual interest is suggested to be a risk factor for all-cause mortality in Japanese males over 40 years old. This finding has implications for the importance of sexual interest in increasing longevity in this population.

## Introduction

Sexual interest is essential for maintaining positive sexual relationships and sexual function, which are recognized as important indicators of good health and quality of life [1–6]. Stulhofer et al. reported that sustained sexual interest and sexual enjoyment are associated with successful aging in both sexes [7, 8]. Lee et al. reported that continuing sexual desire, activity and function are related with higher subjective well-being within the context of a partnered relationship [9]. Uchino et al. also reported that sexual activity was associated with a number of positive psychological factors, including ‘*ikigai*’ and better subjective health [10]. These reports suggest that sexual interest is a positive psychological factor in human life.

Previous studies have shown that positive psychological factors enhance longevity and decrease the risks of cardiovascular disease and cancer [11–14]. Associations have been found between perceived levels of life enjoyment and ‘*ikigai*,’ a Japanese term for a reason for living, or the joy and goal of having a life worth living, and reduced risk of cardiovascular disease and mortality among Japanese people [12]. By contrast, negative psychological factors, including depression, anxiety, as well as psychological distress, are related to increased risk of mortality [15, 16]. From Canadian Community Health Survey Cycle 1.2, subjects with psychological distress had higher mortality rates than those with major depressive disorder [16].

Due to the worldwide increase in the number of aged people, there has been a significant increase in research on sexuality in older persons. Nevertheless, little is known about how sexuality is associated with longevity. We are unaware of any longitudinal prospective study of the association between lack of sexual interest and all-cause mortality, cardiovascular disease mortality, or cancer mortality. Previous studies have found gender-related differences in the effects of psychological factors on longevity, with stronger effects on all-cause mortality, and the incidence of stroke, cardiovascular disease, and cardiovascular disease mortality in men than women [11, 17, 18].

Here, we conducted a prospective evaluation of the relationship between lack of sexual interest and all-cause mortality, cardiovascular mortality, and cancer mortality in both men and women in a community-based population.

## Materials and methods

The Yamagata Study was conducted under a prospective community-based cohort design as one part of a molecular epidemiological study using the regional characteristics of the Global Center of Excellence (COE) program in Japan [19–22]. Participants provided written informed consent prior to enrollment. The study was conducted in accordance with the Declaration of Helsinki. Subjects in this study were patients at an annual community-based health check, and were aged 40 years or older, comprising residents of 7 cities (Kaminoyama, Yamagata, Higashine, Sakata, Tendo and Yonezawa and Sagae) within Yamagata Prefecture, Japan. They were invited to participate to the Yamagata Study. There were no exclusion criteria. From 2009 through 2015, 20,969 people (8,558 males and 12,411 females) were enrolled out of a potential population of 28,528. During follow-up, 541 subjects moved to other areas and were lost to follow-up. The study was approved by the Yamagata University School of Medicine ethics committee (19 Feb 2021, 2020–364).

Participants were followed up to a maximum of 9 years (median, 7.1 years) and evaluated for associations between lack of sexual interest and all-cause mortality, as well as cardiovascular and cancer mortality. We excluded 1,915 subjects from the analysis due to a lack of data at baseline. Finally, data from 19,054 subjects (7,668 males, 11,386 females) were included in the statistical analysis.

### Measurements

At baseline, a self-report questionnaire was mailed to subjects to ascertain their medical history, present use of medications and symptoms, blood pressure, frequency of laughter, sexual interest, smoking status, alcohol consumption, physical activity, marital status, education level, perceived mental stress, as well as involvement in social activities.

Lack of sexual interest was ascertained using a single-item question: “Currently, do you have any interest in people of the opposite sex?” We provided two possible answers, ‘yes’ or ‘no’. Any person who answered ‘no’ was defined as lacking sexual interest. Accordingly, sexual interest in someone of the same sex would be considered as "lacking sexual interest" in this study.

Daily frequency of laughter was assessed via a single-item question: “How often do you laugh out loud?”. Thus, we defined laughter as ‘laughing out loud’. We provided four possible answers: almost every day, 1–5 times/week, 1–3 times/month, and <1 time/month, without allowing free-description responses. Self-reported daily frequency of laughter was grouped into three categories (≥1/week; ≥1/month but <1/week; or <1/month).

Alcohol consumption status was assessed in the three categories of current drinker, past drinker, and nondrinker; the same procedure was used for smoking status. We asked about how often they participated in various civic associations and social groups, classified under the three categories of ≥1/week; ≥1/month but <1/week; and <1/month. Psychological distress was ascertained using the K6, a 6-item instrument used to assess nonspecific psychological distress. Participants with a score of 13 or more points from a total of 24 points were considered to have psychological distress.

Laboratory variables were provided by the health check-up site. Hypertension was classified by a systolic blood pressure ≥140 mmHg or a diastolic pressure of ≥ 90 mmHg, or use of antihypertensive medication. Subjects having a body mass index of ≥ 25.0 kg/m^2^ were classified as obese. The presence of diabetes was ascertained by plasma glucose levels ≥ 126 mg/dL, hemoglobin A1c ≥ 6.5% (National Glycohemoglobin Standardization Program value), or any use of antidiabetic medication. Depression was self-reported as a present or past medical history of depression.

Depression is a mood disorder that manifests as a continuing feeling of sadness or loss of interest. Previous studies have revealed that depression is a risk factor for mortality in the elderly. We also inquired about subjects’ present experience of depression and any medical history of depression, but in view of the small number of reported cases, we omitted this factor from the multivariate analysis.

From the death code certificate, we confirmed the code (International Classification of Disease, 10th Revision), date and location of death. Incidence of cardiovascular mortality was confirmed by reports of the Yamagata Society in Treatment for Cerebral Stroke (YSTCS) and the Yamagata Stroke and Acute Myocardial Infarction (AMI) registry.

### Statistical analysis

Data were expressed using means (plus standard deviation) for continuous values and percentage of total subject number for categorical variables. Analysis of variance was used for comparing differences in mean values, with the chi-squared test used for differences in proportions. Relationships between lack of sexual interest and mortality due to all causes, cardiovascular disease, and cancer were examined by Kaplan-Meier analysis with the log-rank test and unadjusted and adjusted Cox-proportional hazards model analysis. Hazard ratio (HR) with the multivariate-adjusted models were adjusted for age, hypertension, diabetes, smoking, alcohol consumption, BMI, dyslipidemia, education, marital status, frequency of laughter, and psychological distress. Statistical analysis was conducted with JMP v.14 (SAS Institute, Cary, NC, USA).

## Results

Participants were 7,668 males (40.2%) and 11,386 females (59.8%). Mean age was 64.2 and 61.6 years, respectively. Those lacking sexual interest accounted for 8.3% of the male and 16.1% of the female sample. Baseline characteristics of subjects according to sexual interest are shown by gender in Table 1.

**Table 1:** Characteristics of study subjects.

		Male			Female		
		having sexual interest	lack of sexual interest	p-value	having sexual interest	lack of sexual interest	p-value
		n = 7032	n = 636		n = 9551	n = 1835	
Age mean (SD)		64.0 (7.9)	65.9 (7.2)	<0.001**	61.4 (8.7)	62.9 (7.8)	<0.001**
BMI mean (SD)		23.6 (3.0)	23.6 (3.0)	0.754	22.6 (3.3)	22.7 (3.3)	0.183
Obesity		2033 (29.0)	187 (29.4)	0.820	1937 (20.4)	396 (21.6)	0.246
Smoking	Current	1538 (22.5)	165 (26.4)	0.001**	428 (4.8)	105 (6.1)	0.003**
	Past	3554 (51.9)	339 (54.3)		700 (7.9)	168 (9.7)	
	Never	1756 (25.6)	120 (19.2)		7709 (87.2)	1452 (84.2)	
Drinking	Current	5538 (80.1)	483 (76.7)	<0.001**	3542 (38.7)	641 (36.2)	0.016*
	Past	254 (3.7)	46 (7.3)		114 (1.2)	34 (1.9)	
	Never	1126 (16.3)	101 (16.0)		5489 (60.0)	1094 (61.8)	
Medical history, %							
Hypertension		3092 (44.1)	264 (41.5)	0.226	2805 (29.5)	543 (29.6)	0.94
Diabetes		990 (14.4)	117 (18.8)	0.004**	576 (6.3)	128 (7.2)	0.141
Depression		51 (1.5)	9 (2.7)	0.112	99 (2.3)	26 (2.9)	0.408
Dislipidemia		2728 (47.9)	241 (46.6)	0.581	3514 (53.9)	732 (56.9)	0.054
Physical activity,%	≥1 times/week	4556 (69.3)	413 (68.6)	0.828	6236 (70.1)	1237 (70.9)	0.422
	Rarely	968 (14.7)	94 (15.6)		1259 (14.1)	226 (13.0)	
	None	1054 (16.0)	95 (15.8)		1405 (15.8)	281 (16.1)	
Psychological distress (K6≥13)		184(3.0)	33(5.6)	0.001**	382(4.6)	114(6.6)	<0.001**
Frequency of social participation,%	≥1/week	632(10.2)	74(10.4)	0.350	236(2.8)	37(2.1)	0.089
	≥1/month but <1/week	1631(26.4)	159(26.0)		280(17.5)	280(16.1)	
	<1//month	3910(63.3)	378(61.9)		6688(79.7)	1422(81.8)	
Frequency of laughter	≥1 times/week	4781 (75.3)	419 (67.1)	<0.001**	7745 (88.6)	1479 (82.3)	<0.001**
	<1/week but ≥1/month	1244 (19.6)	161 (25.8)		852 (9.8)	254 (14.1)	
	<1/month	322 (5.1)	44 (7.1)		140 (1.6)	65 (3.6)	
Marital status,%	Married	5656 (86.8)	533 (87.2)	0.968	7428 (83.5)	1443 (80.7)	0.002**
	Divorced or Widowed	449 (6.9)	40 (6.5)		1252 (14.1)	308 (17.2)	
	Single	408 (6.3)	38 (6.2)		214 (2.4)	36 (2.0)	
Education, %	primary or junior high school	1229 (18.2)	127 (20.6)	0.014*	1226 (13.3)	265 (14.8)	0.028*
	High school	3705 (54.9)	357 (57.8)		4988 (54.1)	994 (55.5)	
	College or higher education	1812 (26.9)	134 (21.7)		3007 (32.6)	531 (29.7)	

* p<0.05

** p<0.01

Compared to participants who had sexual interest, those who lacked sexual interest included significantly higher percentages who currently smoked, drank in the past, were psychologically distressed, laughed relatively infrequently, and had lower educational attainment. Among men, the rate of diabetes was higher in those who lacked sexual interest than in those who had sexual interest.

**Fig 1:**
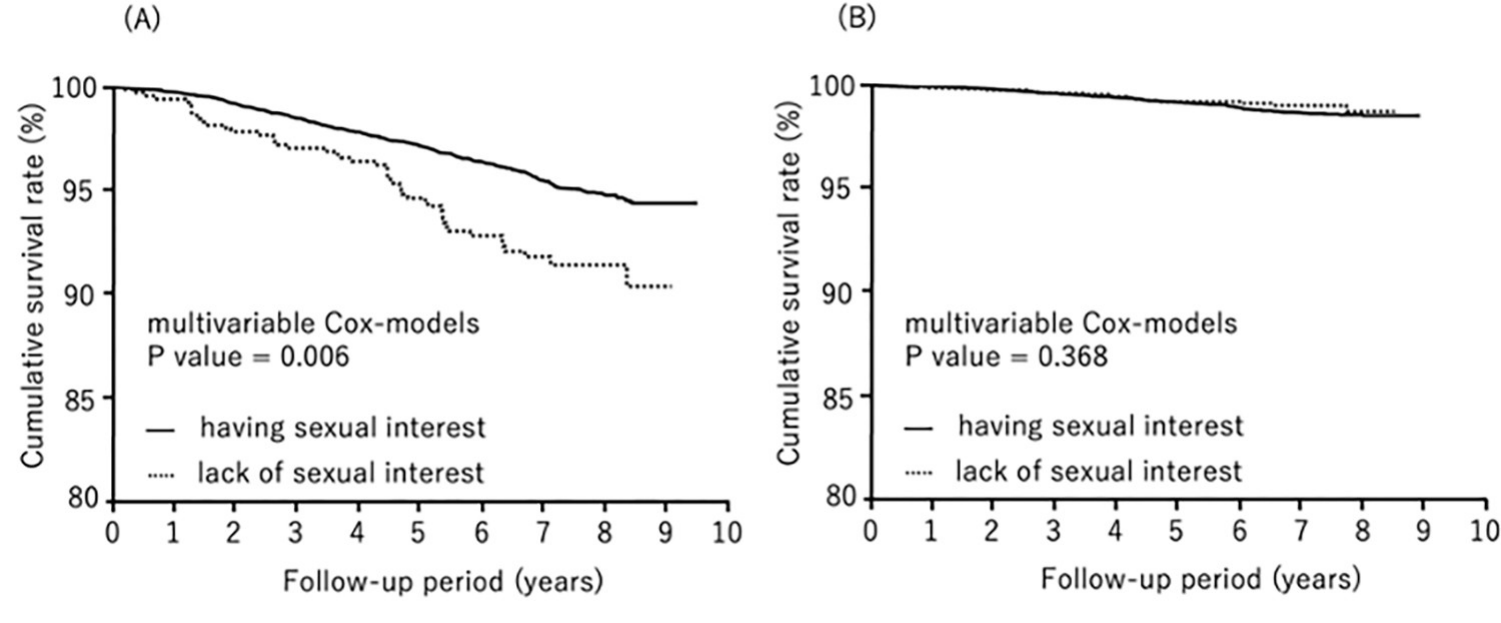
Kaplan-Meier analysis for overall survival by sexual interest. (A) males, (B) females.

**Fig 2:**
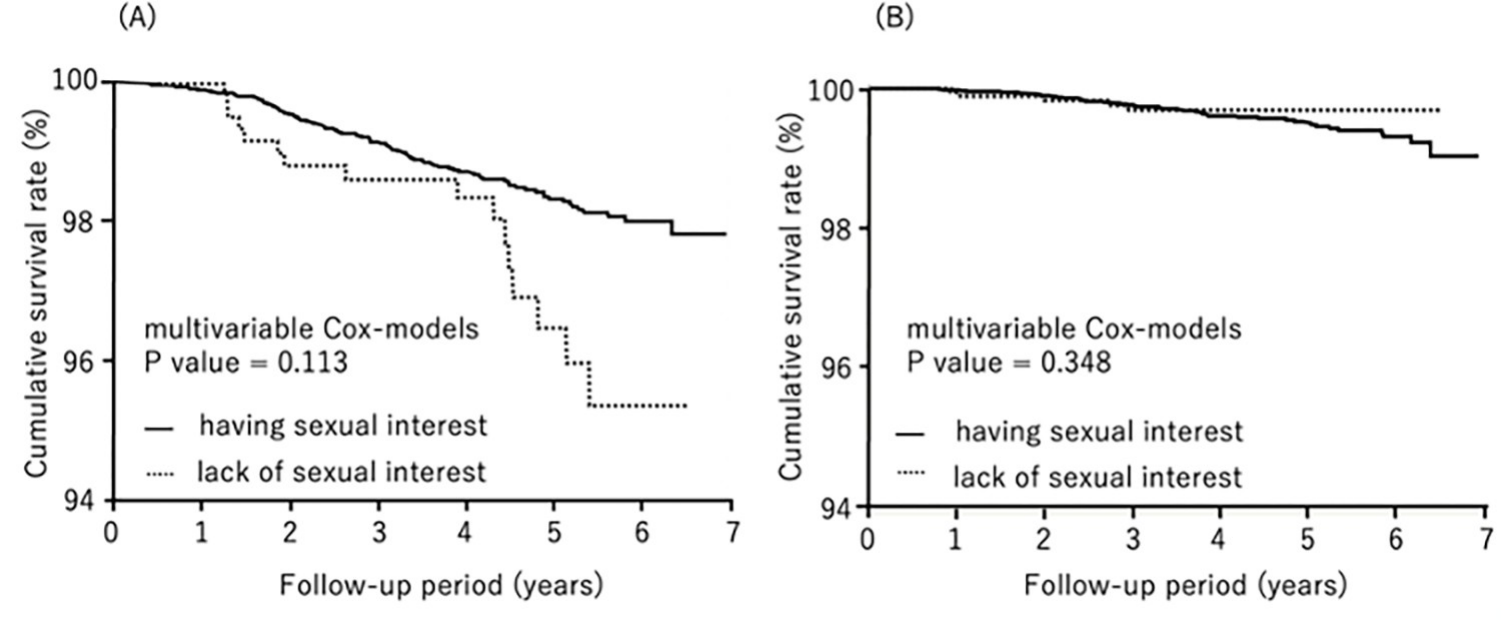
Kaplan-Meier analysis for cancer survival by sexual interest. (A) males, (B) females.

**Fig 3:**
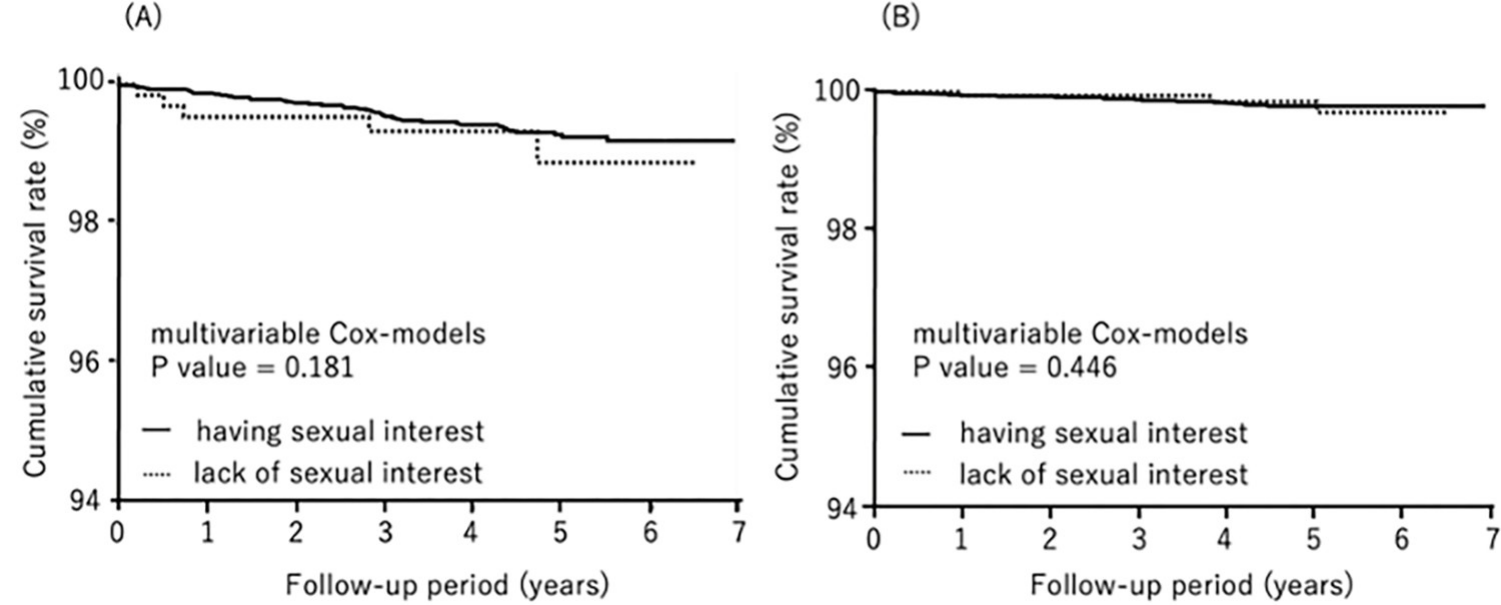
Kaplan-Meier analysis for cardiovascular disease survival by sexual interest. (A) males, (B) females.

During follow-up (median: 7.1 years), 503 subjects died, 67 from cardiovascular disease and 162 from cancer. We compared all-cause mortality, cardiovascular mortality, and cancer mortality by lack of sexual interest and gender by the Kaplan-Meier method. All-cause mortality was significantly higher in males who lacked sexual interest (log-rank test, *P*<0.0001) (Fig 1A). Cancer mortality was also significantly higher in males who lacked sexual interest (log-rank test, *P* = 0.0120) (Fig 2A). There was no statistical effect of lack of sexual interest and cardiovascular mortality in either gender (Fig 3).

We also examined independent associations between lack of sexual interest and all-cause deaths, cardiovascular mortality, and cancer mortality using Cox-proportional hazards analysis (Table 2).

**Table 2:** Sex-specific unadjusted, age-, and multivariable-adjusted HRs and 95% CIs of mortality according to lack of sexual interest.

		Male			Female		
		having sexual interest	lack of sexual interest	p-value	having sexual interest	lack of sexual interest	p-value
All-cause mortality	Person-y	37318.7	4127.4		66814.6	12320.9	
	Cases, n	304	48		127	19	
	Unadjusted HR	1.00	1.82 (1.34–2.47)	<0.001**	1.00	0.81 (0.50–1.31)	0.394
	Age-adjusted HR	1.00	1.63 (1.20–2.21)	0.002**	1.00	0.74 (0.45–1.19)	0.212
	Multivariate HR	1.00	1.69 (1.17–2.44)	0.006**	1.00	0.76 (0.42–1.38)	0.368
Cancer mortality	Person-y	29918.8	2587.0		42440.0	7625.7	
	Cases, n	96	16		44	5	
	Unadjusted HR	1.00	1.94 (1.15–3.31)	0.014*	1.00	0.66 (0.26–1.66)	0.376
	Age-adjusted HR	1.00	1.72 (1.01–2.92)	0.045*	1.00	0.60 (0.24–1.51)	0.278
	Multivariate HR	1.00	1.74 (0.88–3.47)	0.113	1.00	0.60 (0.21–1.74)	0.348
Cardiovascular mortality	Person-y	29918.8	2587.0		42440.0	7625.7	
	Cases, n	42	5		16	3	
	Unadjusted HR	1.00	1.36 (0.54–3.44)	0.512	1.00	1.03 (0.30–3.55)	0.959
	Age-adjusted HR	1.00	1.25 (0.50–3.18)	0.632	1.00	0.94 (0.27–3.22)	0.920
	Multivariate HR	1.00	2.12 (0.70–6.39)	0.181	1.00	1.70 (0.43–6.68)	0.446

* P < 0.05

**P < 0.01

Multivariate adjusted: adjusted for age, hypertension, diabetes, smoking, alcohol consumption, BMI, dyslipidemia, education, marital status, frequency of laughter, and psychological distress

In the unadjusted model, all-cause mortality and cancer mortality were significantly higher among males who lacked sexual interest (HR 1.82; 95% CI, 1.34–2.47, HR 1.94; 95% CI, 1.15–3.31) than those who had sexual interest. After adjustment by age, diabetes, hypertension, dyslipidemia, smoking, alcohol drinking status, BMI, education, marital status, frequency of laughter, and psychological distress, risk of all-cause mortality was significantly greater in males who lacked sexual interest than in males who had sexual interest (HR 1.69; 95% CI, 1.17–2.44). Also in males, lack of sexual interest showed an association with increased risk for cancer mortality following adjustment for age (HR 1.72; 95% CI, 1.01–2.92). However, a statistically significant association was not seen following multivariate adjustment. The absence of sexual interest was not associated with cardiovascular mortality among males. In females, the absence of sexual interest was not associated with all-cause mortality, cardiovascular mortality, or cancer mortality, even in the unadjusted model.

Finally, we investigated the association between absence of sexual interest and incidence of cardiovascular events and cancer. We found no statistically significant associations in the adjusted analysis (data not presented).

## Discussion

Although sexual activity and sexual satisfaction are considered of benefit to psychological health and wellbeing in older groups, the association between sexual interest and longevity has not been investigated. This study is the first to prospectively examine associations between sexual interest and all-cause mortality, and cardiovascular and cancer mortality in a community-based population.

In our sample of people aged 40 years or more, 8.3% of males and 16.1% of females indicated a lack of sexual interest. Similar results were reported by Lindau et al. in two cross-sectional population-based surveys of aging conducted in the US [4]. Baseline characteristics indicated that a lack of sexual interest was associated with greater age, higher prevalence of past alcohol drinking, diabetes, low frequency of laughter, psychological distress, and lower education levels in male subjects. In female subjects, baseline characteristics showed that a lack of sexual interest was associated with greater age, higher prevalence of abstinence from alcohol, low frequency of laughter, psychological distress, divorce or widowhood, and lower education levels. For both sexes, negative psychological variables, including psychological distress and low frequency of laughter, were associated with decreased sexual interest.

Interestingly, our study suggests that lack of sexual interest is associated with all-cause mortality in males, even after adjustment for age, diabetes, hypertension, dyslipidemia, smoking, alcohol intake, BMI, education, marital status, frequency of laughter, and psychological distress. Based on our results, we suggest that lack of sexual interest itself contributes to an increased risk of all-cause mortality, independent of established risk factors in men over 40 years old. However, it is possible that some important confounding factors were not identified or adjusted.

Although to our knowledge the mechanisms underlying the main gender difference result have yet to be clarified, men with ‘*ikigai*’ were at lower risk of cardiovascular mortality than men without ‘*ikigai*,’ subsequent to age and multivariate adjustment. Uzuki et al. found that lack of social support was associated with risk of all-cause mortality and cardiovascular mortality, and these associations were stronger in males than females [22]. Previous studies have shown that risk-reduction effects of positive psychological factors on all-cause mortality and incidence of stroke differed according to gender [11, 17, 18]. Ikeda et al. reported that in Japan men who were divorced or widowed were at higher risk of mortality than married men, whereas no similar trend was observed in women [23]. Based on these results, we speculate that maintaining sexual interest may be related to positive psychological well-being and ‘*ikigai*’ especially among men.

Precisely how a lack of sexual interest impacts on health and longevity remains unknown, although several possibilities can be considered. Male lack of interest may be related with unhealthy lifestyles. In this study, men who reported a lack of sexual interest included more current smokers and cases of diabetes. Furthermore, if we assume that sexual interest is related to positive psychological factors, the absence of interest may affect a range of inflammatory, neuroendocrine, and immune responses. Positive affect is related with reduced neuroendocrine, inflammatory, and cardiovascular activities [24]. Chronic psychological stress has shown an association with suppressed immune response and increased susceptibility to malignancy and infection [25]. Further study is required to clarify the mechanisms which underlie the preventive effects of sexual interest on mortality.

The strengths of this study are its prospective design and substantial sample size. In addition, adjustment was done using a number of well-known risk factors, including age, diabetes, hypertension, dyslipidemia, smoking status, alcohol intake, psychological distress, and medical history of depression. However, several limitations of the study should be noted. First, although we conducted multivariate analyses with adjustment for various potential confounding factors, some unidentified confounding factors may have remained. Moreover, we did not adjust for other medically relevant elements known to affect sexual function and longevity, such as neurological conditions, depression, and medications, because such information was not obtained in the baseline survey. Also, only a small number of depressed patients were included in this study. Second, we did not consider how the social regulation of sexuality differs among cultures. Similar research should be extended to other countries. Third, our question about lack of sexual interest focused on interest in the opposite sex; we did not control for sexual orientation.　Sasayama et al. reported that the prevalence of homoromantic attraction was 1.0% for females and 1.5% for males [26]. Based on these figures, the potential number of individuals in the present study with sexual interest in the same sex was estimated to be approximately 200 people. We therefore consider it unlikely that this number would have influenced our overall results. We suggest that future research should include lesbian, gay, bisexual, transgender, and questioning (LGBTQs) adults. Fourth, this study collected data on subjects who were at least 40 years old. Future research might also look more closely at the onset of decreasing interest in sex; for example, whether these results concern only men who had recently lost interest, or whether they extend to men who had little interest even when they were much younger. Fifth, all subjects participated in community-based annual health checks, and so they might have been more health-conscious and more socially active than the general population. In other words, some degree of selection bias might characterize our study sample.

Notwithstanding these limitations, the findings of this study support that idea that maintaining sexual interest has positive effects on longevity, especially in males. The Canadian government, through public health promotion materials, has begun to endorse sexual activity as one element of an “aging well” agenda [27]. In Japan, there is more prejudice about sex among the elderly than in the Western world. We hope our findings will help promote public health through advocating sexuality in Japan.

## Conclusion

This study suggested that lack of sexual interest is a risk factor for all-cause mortality in Japanese males but not females. These findings suggest that maintaining sexual interest might increase longevity in males.
